# Genetic characterization of EV71 isolates from 2004 to 2010 reveals predominance and persistent circulation of the newly proposed genotype D and recent emergence of a distinct lineage of subgenotype C2 in Hong Kong

**DOI:** 10.1186/1743-422X-10-222

**Published:** 2013-07-04

**Authors:** Cyril CY Yip, Susanna KP Lau, Janice YC Lo, Kwok-Hung Chan, Patrick CY Woo, Kwok-Yung Yuen

**Affiliations:** 1 Department of Microbiology, The University of Hong Kong, Hong Kong, China; 2 State Key Laboratory of Emerging Infectious Diseases, The University of Hong Kong, University Pathology Building, Queen Mary Hospital, Hong Kong, China; 3 Research Centre of Infection and Immunology, The University of Hong Kong, Hong Kong, China; 4 Carol Yu Centre for Infection, The University of Hong Kong, Hong Kong, China; 5 Centre for Health Protection, Department of Health, Hong Kong, China

**Keywords:** Enterovirus 71, Hand foot and mouth disease, Recombination, Lineage, Epidemics, Hong Kong

## Abstract

**Background:**

Enterovirus 71 (EV71) is a common etiological agent of hand, foot and mouth disease (HFMD) in children. EV71 epidemics have been reported in Hong Kong in recent years, and yet the genetic information of EV71 strains circulating in our locality is limited. The objective of this study was to investigate the genetic evolution of these EV71 isolates in Hong Kong over a 7-year period.

**Methods:**

Twenty-two EV71 isolates from Hong Kong during 2004–2010 were included for phylogenetic analysis using partial VP2-VP3, 2C and 3D regions. Eight EV71 strains were selected for complete genome sequencing and recombination analysis.

**Results:**

Among the 22 EV71 isolates, 20 belonged to subgenotype C4 and 2 belonged to subgenotype C2 based on the phylogenetic analysis of partial VP2-VP3, 2C and 3D gene regions. Phylogenetic, similarity plot and bootscan analyses using complete genome sequences of seven EV71 isolates of subgenotype C4 supported that the “double-recombinant” strains of subgenotype C4 persistently circulating in Hong Kong should belong to a newly proposed genotype D. Further analysis revealed two clusters, subgenotypes C4b and C4a (proposed genotypes D1a and D1b respectively), with “genotype D1b” strains being predominant in recent years in Hong Kong. A distinct lineage of EV71 subgenotype C2 has emerged in Hong Kong in 2008. The evolutionary rate of EV71 was 3.1 × 10^-3^ nucleotide substitutions per site per year similar to that of other enterovirus, such as EV68, but was relatively lower than those of echovirus 30 and poliovirus. Molecular clock analysis using VP1 gene dated the time to the most recent common ancestor of all EV71 genotypes to 1900s, while the EV71 “double-recombinant” strains of “genotype D” were detected as early as 1998.

**Conclusions:**

This study provides the molecular basis for proposing a new “genotype D” of EV71 and assigning a discrete lineage of subgenotype C2. EV71 strains of “genotype D” have been circulating in Hong Kong for over 7 years, with “genotype D1b” being predominant.

## Background

Enterovirus 71 (EV71) belongs to human enterovirus species A (HEV-A) in the genus *Enterovirus* of the family *Picornaviridae*[1]. While being a common causative agent of hand, foot and mouth disease (HFMD) and herpangina in young children, it can cause severe complications, such as myocarditis, acute flaccid paralysis, aseptic meningitis, encephalitis and even death [1-3]. EV71 is classified into three genotypes A, B and C based on molecular analysis using VP4 and VP1 gene sequences [4]. The prototype EV71 strain BrCr belonging to genotype A was first identified in California in 1970 [5,6], and this genotype was not detected thereafter until 2008 when genotype A strains re-emerged in central China [7]. Genotypes B and C are each further divided into five genotypes B1-B5 and C1-C5, respectively [4,8]. A research group has proposed a novel subgenotype B0 of EV71 that was circulating in the Netherlands from 1963 to 1967 [9].

Mutation and recombination are common phenomena in the evolution of enteroviruses [10-12]. The infidelity of enterovirus 3D polymerase can result in a mutation rate as high as one mutation per every newly synthesized genome [13]. The preferential recombination in non-structural protein coding regions, where a high nucleotide sequence identity between two parental strains may favor template switching during negative strand synthesis, is thought to be mediated by a “copy-choice” mechanism [12,14]. The occurrence of intratypic and intertypic recombination events in EV71 has been reported during EV71 epidemics in Asian countries since the late 1990s [15-19]. A study from Taiwan revealed that intratypic recombination between EV71 genotypes B and C has taken place in one EV71 isolate N3340-TW-02 [18]. In China, intertypic recombination between EV71 subgenotype C2 and coxsackievirus A16 (CVA16) strain G-10 has occurred in two EV71 isolates, SHZH98 and SHZH03 [20]. In 2008, we have performed complete genome sequencing on two EV71 strains, SZ/HK08-5 and SZ/HK08-6, from Shenzhen during a large HFMD outbreak in China and the result showed that both intra- and inter-typic recombination events were detected in the two strains [15]. These unique “double-recombinant” and other subgenotype C4 strains were proposed to belong to a new genotype, genotype D. Another research group also demonstrated that subgenotype C4 strains should be designated as genotype D [19]. EV71 epidemics have been detected in Hong Kong [21], but the molecular epidemiology and genomic data of EV71 isolates circulating in our population is lacking. In this study, we aim to examine the evolutionary pattern of EV71 over a 7-year period in Hong Kong. EV71 isolates detected from 22 patients in Hong Kong during 2004–2010 were subject to partial VP2-VP3, 2C and 3D gene sequencing and analysis. Since initial phylogenetic analysis using the partial gene regions suggested the presence of potential recombination events in EV71 of subgenotype C4 (proposed genotype D) and a separate lineage of subgenotype C2 in Hong Kong, we performed complete genome sequencing on eight EV71 strains and complete VP1 gene sequencing on 22 EV71 isolates in Hong Kong for further analysis.

## Results

### Clinical characteristics of patients with EV71 infections

EV71 isolates were detected from 17 stool, 2 nasopharyngeal aspirate (NPA), 2 throat swab and 1 rectal swab samples of 22 patients in Hong Kong. Most patients were young children (median age 3 years, ranged from 9 months to 32 years) who presented with HFMD without any complications. Ten were males and 12 were females. The clinical characteristics of the 22 patients with EV71 infections were summarized in Table 1.

**Table 1:** Clinical characteristics and demographic data of 22 cases of EV71 infections

**Strain**	**Sample**	**Collection date**	**Gender**	**Age**	**Diagnosis**	**Genotype**^**#**^
V04-2216042	Stool	30-4-2004	F	3	HFMD	D
V04-2218217*	Stool	18-5-2004	M	2	HFMD	D
V04-2223605	TS	28-6-2004	F	9	HFMD	D
V05-2243055*	Stool	8-9-2005	F	3	HFMD	D
V05-2243936	Stool	14-9-2005	F	3	HFMD	D
V06-2218645*	Stool	26-4-2006	F	10	HFMD	D
V06-2223557	Stool	25-5-2006	F	15	HFMD	D
V06-2224881	Stool	2-6-2006	M	14	Suspected HFMD	D
V07-2231013*	Stool	23-5-2007	M	4	HFMD	D
V07-2232477	Stool	31-5-2007	M	3	HFMD	D
V07-2233174	TS	5-6-2007	M	5	HFMD	D
V08-2221581*	RS	14-4-2008	M	2	HFMD	D
V08-2228220	Stool	19-5-2008	F	1	HFMD	D
V08-2235341	Stool	26-6-2008	F	3	HFMD	D
V08-2231530	Stool	5-6-2008	F	4	NA	C2
V08-2236079*	Stool	30-6-2008	F	3	HFMD	C2
V09-2220211	Stool	8-4-2009	F	2	Suspected HFMD	D
V09-2225777*	Stool	6-5-2009	M	2	HFMD	D
V09-2229604	NPA	25-5-2009	M	2	HFMD	D
V10-2234054*	Stool	19-4-2010	M	32	HFMD	D
V10-2240501	Stool	10-5-2010	F	9m	NA	D
V10-2246239	NPA	1-6-2010	M	1	HFMD	D

*: EV71 strains subject to complete genome sequencing.

^#^: subgenotype C4 proposed to be genotype D.

Abbreviations: *TS* throat swab, *RS* rectal swab, *NPA* nasopharyngeal aspirate, *HFMD* hand, foot and mouth disease, *NA* not available.

### RT-PCR and sequencing of partial VP2-VP3, 2C and 3D gene regions of EV71 and phylogenetic analysis

**Figure 1:**
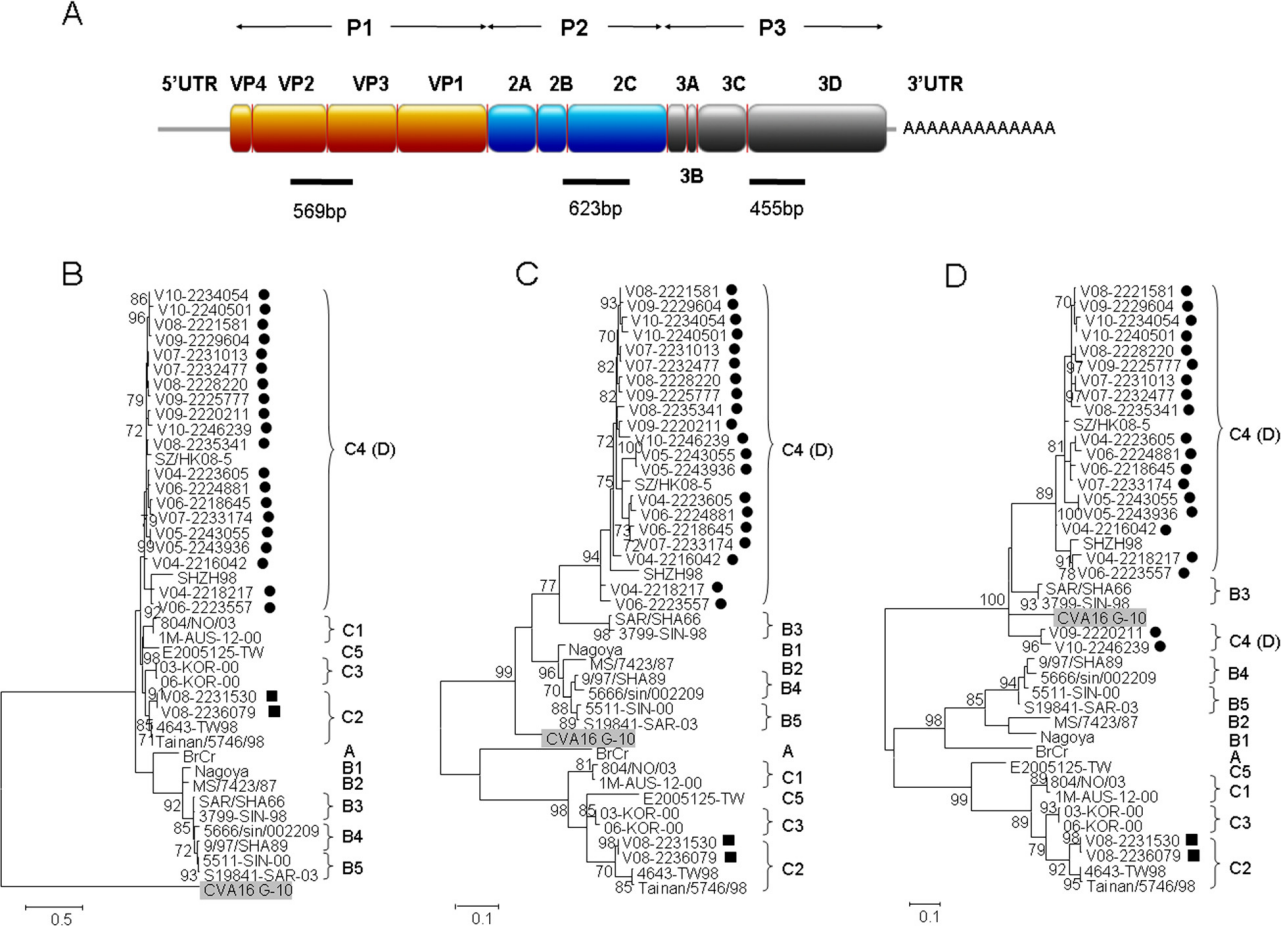
**Schematic representation of EV71 genome (A).** Partial VP2-VP3, partial 2C and partial 3D regions used for phylogenetic analysis are indicated by black bars. Phylogenetic trees of partial VP2-VP3, 2C and 3D regions of EV71 and CVA16 **(B-D)**. The trees were inferred from partial VP2-VP3 **(B)**, partial 2C **(C)** and partial 3D **(D)** region data by maximum likelihood (ML) method, with bootstrap values calculated from 1000 trees. Sequences for 529 nucleotide positions in each partial VP2-VP3 region, 583 nucleotide positions in each partial 2C region and 415 nucleotide positions in each partial 3D region were included in the analysis. Bootstrap values expressed as percentage are shown at the nodes and the scale reflects the number of nucleotide substitutions per site along the branches. Only bootstrap values of >70% are shown. Black circles and squares indicate EV71 isolates of subgenotype C4 (proposed genotype D) and those of subgenotype C2 respectively from Hong Kong in the present study. CVA16 prototype strain G-10 is highlighted in grey.

The partial VP2-VP3, 2C and 3D gene regions of EV71 from the 22 viral isolates were amplified and sequenced (Figure 1A). Based on the phylogenetic analysis using partial VP2-VP3 gene sequences (Figure 1B), 20 isolates were clustered with EV71 subgenotype C4 strains and 2 were closely related to EV71 subgenotype C2 strains. Further analysis showed that the 20 EV71 subgenotype C4 strains were clustered with EV71 genotype B strains for partial 2C region (Figure 1C), but with EV71 subgenotype B3 strains and CVA16 prototype strain G-10 for partial 3D region (Figure 1D). Such incongruent phylogenetic relationships observed among different partial gene regions of our 20 EV71 subgenotype C4 strains suggested that these strains may belong to a newly proposed genotype D, which may have arisen from intra- and inter-typic recombinations as described previously [15]. On the other hand, the two EV71 subgenotype C2 strains, V08-2231530 and V08-2236079, were clustered with other subgenotype C2 strains for partial 2C and 3D regions (Figure 1C and 1D), indicating that they belonged to subgenotype C2. The result showed that except the two EV71 strains belonging to subgenotype C2, all EV71 strains from Hong Kong during 2004–2010 included in this study belonged to subgenotype C4 (proposed genotype D), revealing that “genotype D” was the predominant genotype persistently circulating in our population.

### Complete genome analysis of EV71 strains from Hong Kong

To confirm if recombination events have occurred in EV71 strains from Hong Kong, 7 “genotype D” strains and 1 subgenotype C2 strain were selected for complete genome sequencing (Table 1). The sizes of the genomes of the eight EV71 strains ranged from 7404 to 7409 nucleotides, with G + C content of 47.5 to 48.6% (excluding the 3′ polyA tract). Their genome organization was typical of *Enterovirus* (Figure 1A). The complete genome sequences of the 7 “genotype D” strains were highly similar, with the nucleotide sequence identities of 92.7-98.3%. Using 7 genome sequences of the “genotype D” strains from Hong Kong for analysis, the Ka/Ks ratios for the various genes were calculated (Additional file 1: Table S1). The Ka/Ks ratios of all genes were small, implying that these genes in structural and non-structural protein coding regions were stably evolving over 7 years.

**Figure 2:**
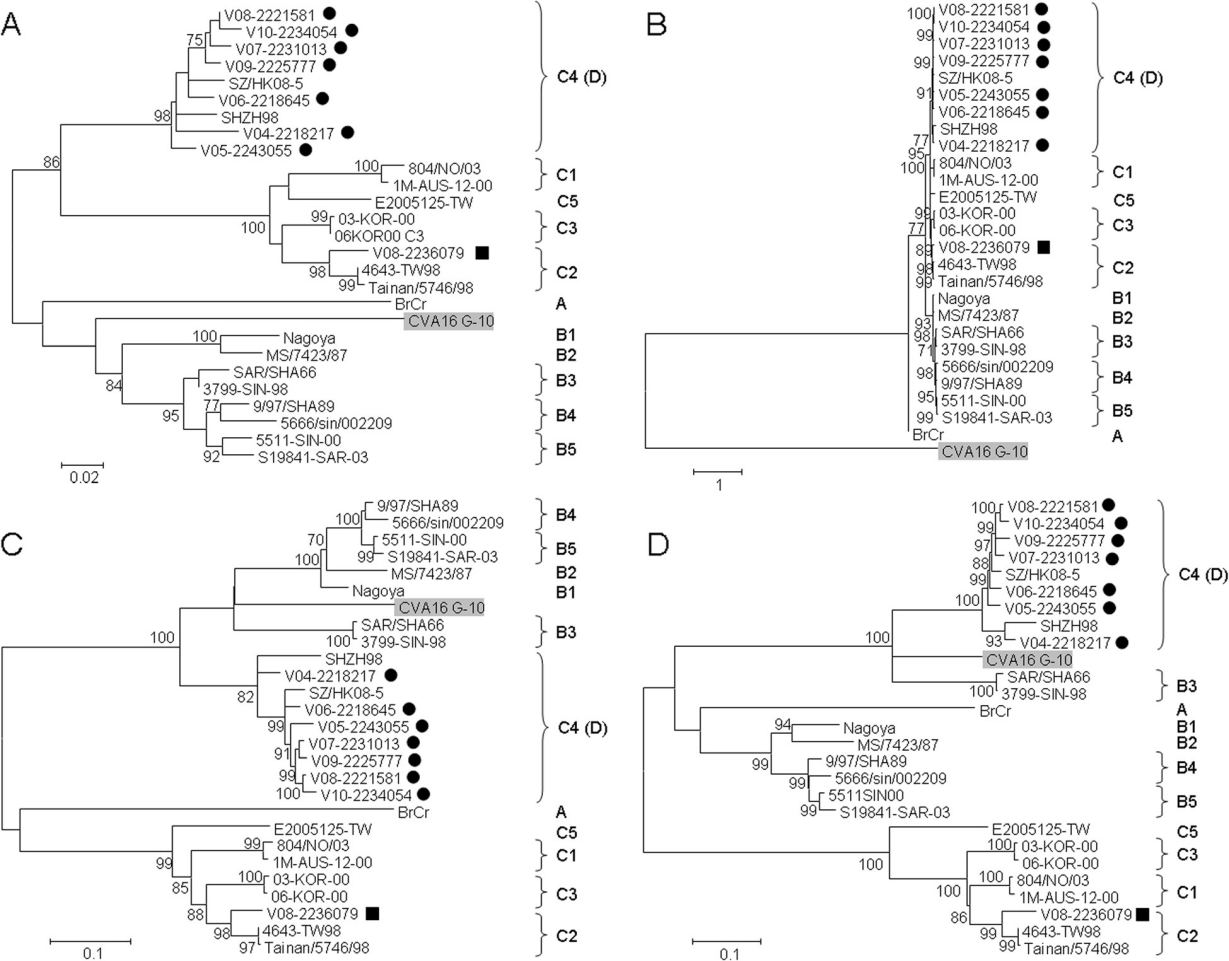
**Phylogenetic trees of 5'UTR, P1, P2 and P3 regions of EV71 and CVA16 strains with complete genome sequences.** The trees were inferred from 5′UTR **(A)**, P1 **(B)**, P2 **(C)** and P3 **(D)** region data by ML method, with bootstrap values calculated from 1000 trees. Sequences for 751 nucleotide positions in each 5′UTR, 2586 nucleotide positions in each P1 region, 1734 nucleotide positions in each P2 region and 2259 nucleotide positions in each P3 region were included in the analysis. Black circles and squares indicate EV71 isolates of subgenotype C4 (proposed genotype D) and those of subgenotype C2 respectively from Hong Kong in the present study. CVA16 prototype strain G-10 is highlighted in grey.

Phylogenetic trees using nucleotide sequences of 5′ untranslated region (5′UTR), P1 (VP4 to VP1), P2 (2A to 2C) and P3 (3A to 3D) regions of 7 “genotype D” and 1 subgenotype C2 strains from Hong Kong and other EV71 strains with complete genome sequences available in the GenBank were constructed (Figure 2). Phylogenetic analysis showed that the EV71 subgenotype C2 strain V08-2236079 was most closely related to other subgenotype C2 strains for all four regions, which was in line with the result of bootscan analysis (data not shown), suggesting that no recombination has occurred in subgenotype C2 strains in Hong Kong in this study. While the phylogenetic analysis revealed that the seven EV71 “genotype D” strains were clustered with EV71 genotype C strains for 5′UTR and P1 region, but with EV71 genotype B strains and CVA16 strain G-10 for P2 and P3 regions, respectively.

**Figure 3:**
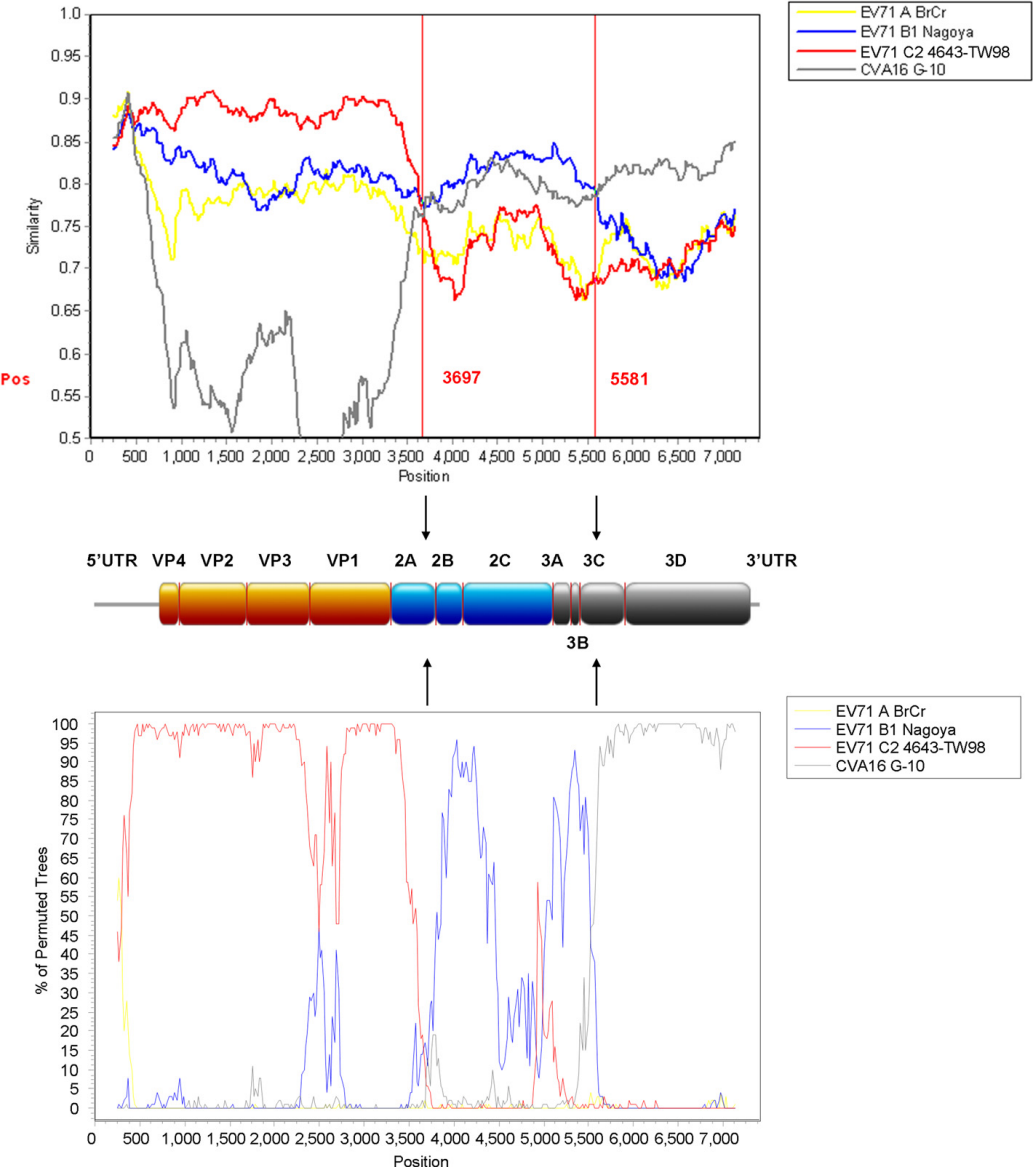
**Recombination analysis of the complete genome of EV71.** Similarity plot (upper panel) and bootscan analysis (lower panel) were conducted with SimPlot version 3.5.1 (Kimura distance model; window size, 500 bp; step, 20 bp) on a gapless nucleotide alignment, generated with ClustalX, with the genome sequence of the EV71 “genotype D” strain V10-2234054 as the query sequence. The yellow line denotes EV71 genotype A strain BrCr, the blue line denotes EV71 genotype B strain Nagoya, the red line denotes EV71 genotype C strain 4643-TW98, and the grey line denotes CVA16 prototype strain G-10. Arrows indicate the gene regions in which potential recombination breakpoints are located.

Similarity plot and bootscan analyses, using an EV71 “genotype D” strain V10-2234054 as a query sequence, were performed to identify potential recombination sites (Figure 3). The analyses demonstrated that two recombination sites were probably located at 2A-2B junction (between nucleotide positions 3500 and 3800) and 3C region (between positions 5500 and 5600) (Additional file 2: Figure S1), which were similar to those identified in the two EV71 strains from Shenzhen as described earlier [15]. The region upstream of 2A-2B junction of the strain V10-2234054 was most closely related to a genotype C strain, but the region between the two breakpoints was highly similar to a genotype B strain, while the region beyond the breakpoint at 3C shared higher similarity with CVA16 strain G-10. Similar recombination patterns were also observed in other 6 EV71 “genotype D” strains in Hong Kong (data not shown). The above findings suggested that all EV71 strains from Hong Kong in this study, except strains V08-2231530 and V08-2236079, belonged to the double recombinant “genotype D”.

### Complete VP1 gene sequence analysis

**Figure 4:**
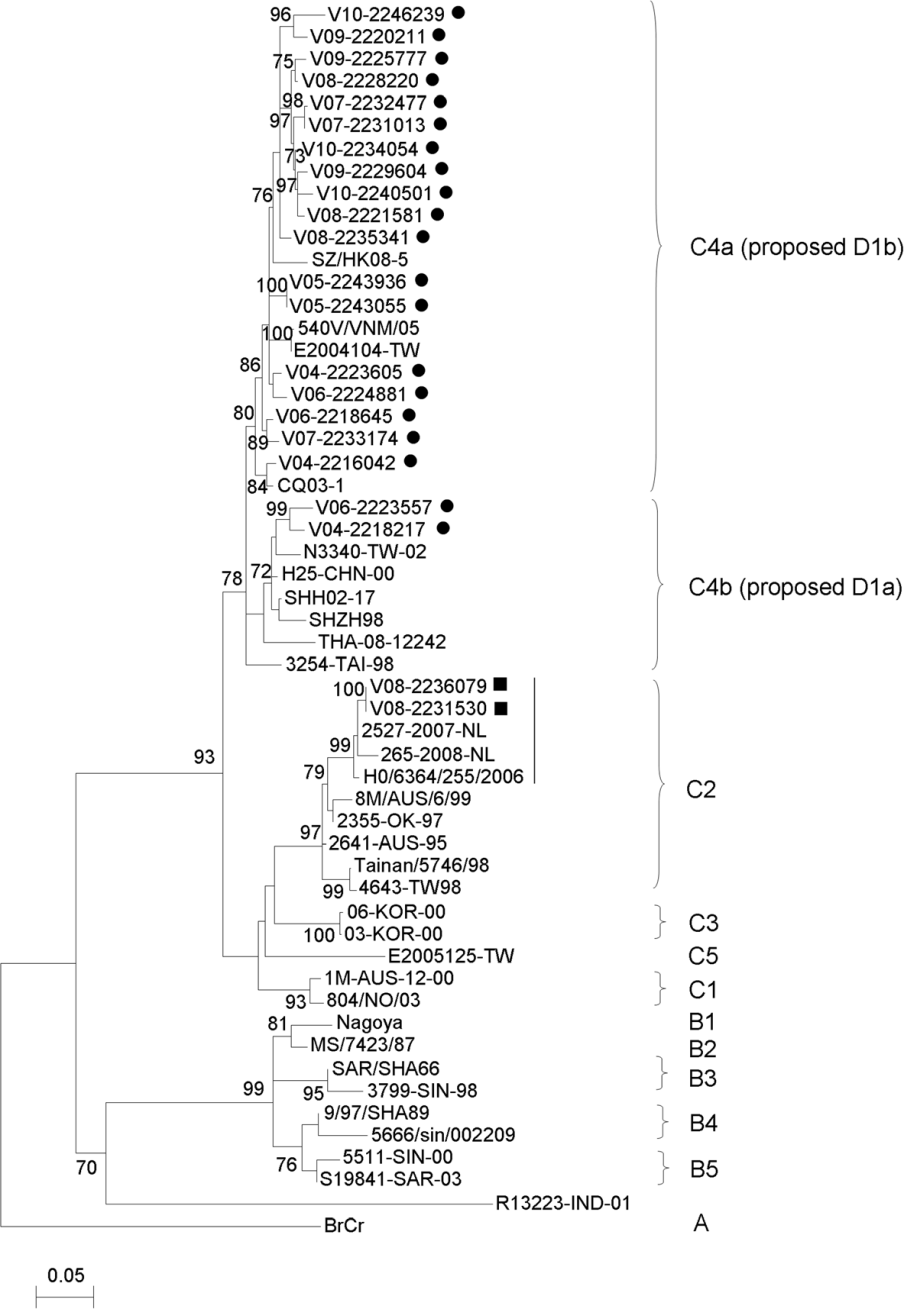
**Phylogenetic analysis using VP1 gene sequences (855 bp) of EV71.** The tree was constructed using ML method, with bootstrap values calculated from 1000 trees. Black circles and squares indicate EV71 isolates of subgenotype C4 (proposed genotype D) and those of subgenotype C2 respectively from Hong Kong in the present study. A vertical black line indicates a potential distinct lineage of EV71 subgenotype C2 strains.

The complete VP1 sequences of 22 EV71 isolates in Hong Kong were amplified and sequenced. Phylogenetic analysis showed that 20 EV71 strains were clustered with other EV71 strains of subgenotype C4 (proposed genotype D) (Figure 4). Among these 20 EV71 strains, 18 belonged to subgenotype C4a (proposed genotype D1b), while 2 belonged to subgenotype C4b (proposed genotype D1a). A nucleotide divergence of 5.9-9.3% was observed between “genotype D1a” and “genotype D1b” strains from Hong Kong. Furthermore, the nucleotide divergence of the subgenotype C4 strains is 8.1-14% when compared to other subgenotype C strains, while that of the subgenotype C4 strains is 14.8-18.2% when compared to EV71 genotype B strains.

Besides, the phylogenetic analysis revealed that two EV71 strains, V08-2231530 and V08-2236079, belonged to subgenotype C2 (Figure 4). Intriguingly, these two EV71 strains from Hong Kong and other subgenotype C2 strains detected in recent years (2006–2008) had a high degree of VP1 nucleotide sequence identity of 97.6-100% and formed a cluster distinct from subgenotype C2 strains found in the 1990s (Figure 4). In addition, a nucleotide divergence of 2.7-6.5% was found between the “old” and “new” subgenotype C2 strains. These findings suggested that the recent subgenotype C2 strains may represent a new lineage, which has emerged in Hong Kong since 2008.

### Estimation of divergence dates

**Figure 5:**
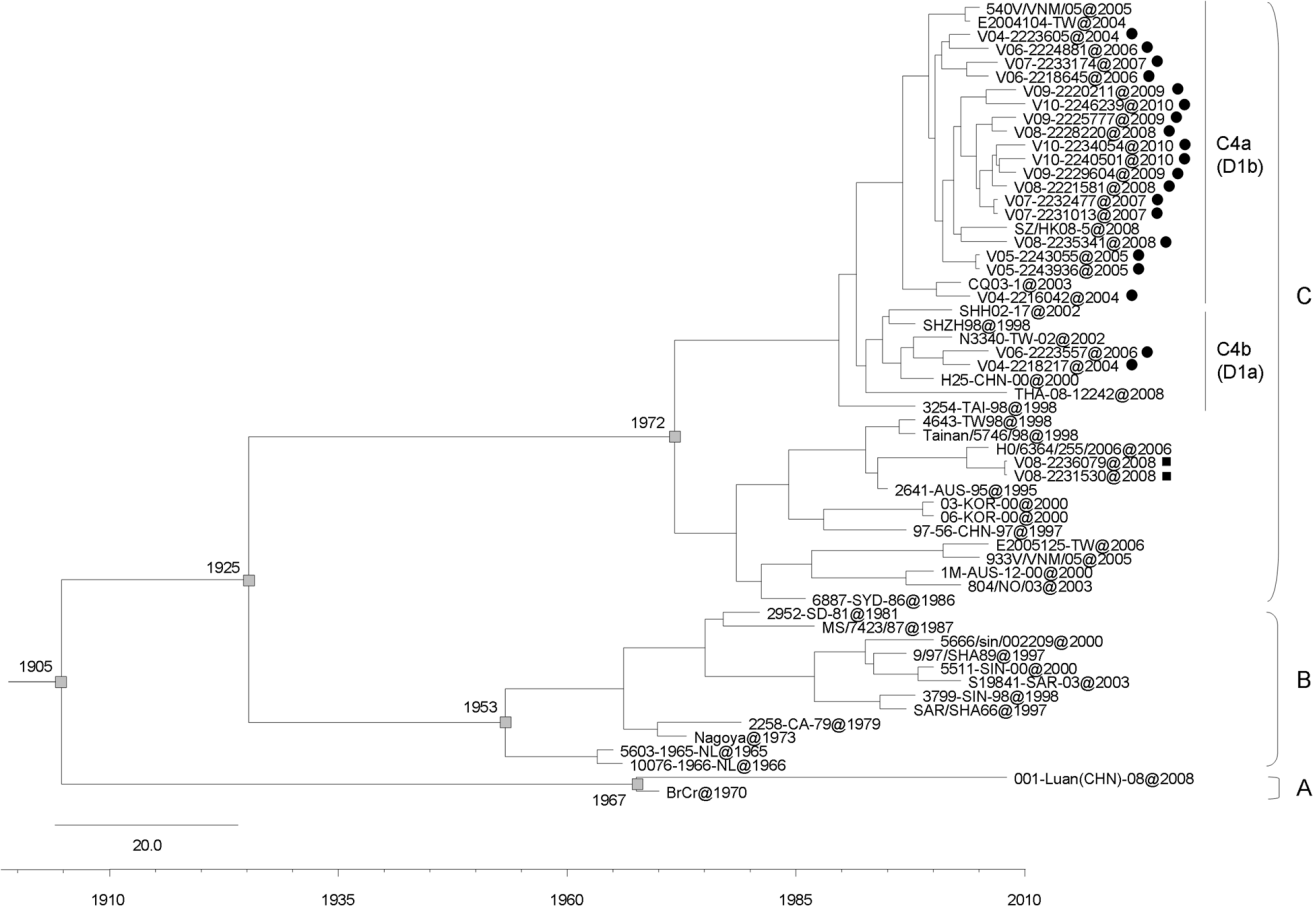
**Estimation of the tMRCA for EV71 genotypes based on the VP1 gene.** The mean estimated dates are labeled and are represented by grey squares. The taxa are labeled with their sampling dates. **A**, genotype A; **B**, genotype B and **C**, genotype C. Subgenotypes C4b and C4a are proposed to be genotypes D1a and D1b respectively. Black circles and squares indicate EV71 isolates of subgenotype C4 (proposed genotype D) and those of subgenotype C2 respectively from Hong Kong in the present study.

Using the exponential growth population size coalescent under a relaxed clock model with an uncorrelated lognormal distribution, the mean evolutionary rate of all EV71 genotypes was 3.1 × 10^-3^ nucleotide substitutions per site per year (s/s/y) for VP1 gene, which was consistent with a previous finding of 3.28 × 10^-3^ s/s/y [22]. Molecular clock analysis using VP1 gene showed that the time to most recent common ancestor (tMRCA) of all EV71 genotypes A, B and C was estimated at 1905 (HPDs, 1858 to 1943), 105 years ago; that of genotypes B and C at 1925 (HPDs, 1895 to 1951), 85 years ago; that of genotype B at 1953 (HPDs, 1942 to 1963), 57 years ago; that of genotype A at 1967 (HPDs, 1964 to 1970), 43 years ago; and that of genotype C at 1972 (HPDs, 1960 to 1982), 38 years ago (Figure 5). These estimates were consistent and were also in line with a previous study showing the tMRCA of EV71 strains at 1911 [22].

## Discussion

The present study is the first report of the emergence of EV71 “double-recombinant” strains belonging to the newly proposed genotype D in Hong Kong. We have previously showed that intratypic and intertypic recombination events have occurred in EV71 strains, SZ/HK08-5 and SZ/HK08-6, and other subgenotype C4 strains, which represent the new genotype D [15]. Although the functional relevance of the “double recombination” events in EV71 remains unclear, we speculate that the acquisition of replicative components, such as helicase, protease and polymerase, from EV71 genotype B strain and CVA16 strain in EV71 subgenotype C4 strain may confer evolutionary advantages on EV71 “double-recombinant” strains of “genotype D”. Further studies are warranted to investigate the role of the recombinations in the “genotype D” strains. In the present study, the incongruent phylogenetic relationships among the 5′UTR, P1, P2 and P3 regions of the subgenotype C4 strains, together with results of similarity plot and bootscan analyses, confirmed that these strains belonged to the “double-recombinant”, newly proposed genotype D. Such incongruities observed in EV71 phylogenetic trees when comparing structural and non-structural regions of the genomes were similar to those demonstrated in a recent study [23], which showed that a mosaic of incongruent patterns for EV71 were likely due to the independent evolutionary pathways of the two genome regions of EV71. Traditionally, many studies have reported the use of structural gene sequences [4,24,25], particularly VP1 gene, for EV71 genotype and subgenotype determination. However, VP1 gene sequence mainly provides information on the serotype of EV71 and it encompasses only about one-tenth of the entire genome. On the other hand, using whole genome sequence for EV71 genotyping seems to be more appropriate because it consists of not only structural region encoding sequences, but also non-structural region encoding sequences and untranslated regions at 5′ and 3' termini. In addition, recombination commonly occurred in enteroviruses [10-12,15], such as EV71 subgenotype C4 strains, which were shown to cluster with other subgenotype C strains for 5′UTR and P1, but not for P2 and P3 regions (Figure 2). Furthermore, in other positive-sense single-stranded RNA viruses, such as human coronaviruses (HCoVs), the generation of novel genotypes of HCoV-HKU1 and HCoV-OC43 as a result of the recombination between different genotypes has been reported [26,27]. Renaming subgenotype C4 as genotype D was also supported by another study [19], in which the complete genome sequences of subgenotype C4 strains were shown to have a nucleotide divergence of 17-20% that exceeded the average cut-off divergence of 14.95% for subgenotyping. Similar nucleotide divergence (17–18.9%) of the 7 subgenotype C4 strains in the present study was also demonstrated when compared to other EV71 strains of subgenotype C1, C2, C3 and C5 using complete genome sequences. A combination of VP1 and 3D gene sequences was proposed to be used for initial genotyping of EV71 isolates in the absence of complete genome sequences [19]. In 2001, an EV71 isolate from a patient with acute flaccid paralysis in India has also been proposed to belong to genotype D [28,29]. However, complete genome sequence data were lacking for more detailed phylogenetic and genome analysis of this Indian strain. As VP1 gene clustering closely resembles the serotype designation of enteroviruses, VP1 gene should be sequenced for EV71 genotyping in any circumstances. However, using VP1 gene alone may be insufficient for genotyping the EV71 recombinants. Thus, sequencing of at least three regions, probably one from P1, one from P2 and one from P3, is only required for genotyping the EV71 strains with recombination, and this will help future assignment of genotypes for enteroviruses.

EV71 “genotype D” strains have been persistently circulating in Hong Kong, which has experienced two EV71 epidemics in 2008 and 2010 with around 100 reported cases each year [21,30]. Among the 22 EV71 isolates collected in this study, 20 belonged to “genotype D” and two belonged to subgenotype C2, indicating that “genotype D” was the predominant type of EV71 in our locality over the 7-year period. This is consistent with an epidemiological study showing that subgenotype C4 (“genotype D”) was the major type of EV71 circulating in Hong Kong since 1998 [21]. Persistent circulation of subgenotype C4 strains was also noted in China during 1998–2010 [31]. We found that the complete genome sequences of the 7 subgenotype C4 strains in Hong Kong were highly similar to those in China, with nucleotide sequence identity of >90%. As Hong Kong is well connected to China, this may facilitate the spread of subgenotype C4 strains from mainland China to Hong Kong. The reasons for the recent EV71 epidemics in Hong Kong due to the same genotype of virus, “genotype D”, remain to be determined. One possible explanation is that immune response against EV71 in individuals may decrease over time, leading to an accumulation of susceptible individuals, in turn facilitating the transmission of EV71 “genotype D” in our population. Further study is warranted to determine if lower seroprevalence is noted in a year prior to each EV71 epidemic. Another reason for a surge in the number of EV71 cases could be owing to the antigenic changes of EV71 [17]. However, when comparing the amino acid sequences of VP1 of the 20 “genotype D” strains from Hong Kong, they were highly identical with conserved amino acid residues in the BC loop essential for interaction with neutralizing antibodies. The high degree of conservation in the immunogenic VP1 protein of “genotype D” strains raises the possibility of developing a vaccine against this predominant genotype in Hong Kong and mainland China, which may help protect young children at high risk.

As EV71 subgenotype C4 should be re-designated as genotype D, we propose to change subgenotypes C4b and C4a into genotypes D1a and D1b respectively. The earliest characterized subgenotype C4 strain SHZH98 is in cluster b, but for more systematic classification (usually based on chronological order), the older and recent strains should belong to cluster a and b respectively. Since the late 1990s, EV71 subgenotype C4 strains have been frequently reported in Asian countries [22,32-37]. The subgenotype C4 was further divided into two clusters, C4b and C4a [22,37], which are better to be renamed as genotypes D1a and D1b respectively. In the present study, EV71 “genotype D” strains in Hong Kong have been stably evolving during 2004–2010, with a shift from “genotype D1a” (2004 and 2006) to “genotype D1b” (2004–2010). From the results of the present study, no correlation was found between genotypes of EV71 and disease severity, though a recent study from China showed that C4a strains (proposed genotype D1b) caused more severe diseases in patients with HFMD than C4b strains (proposed genotype D1a) [22].

In addition to “genotype D”, a distinct lineage of EV71 subgenotype C2 has emerged in Hong Kong in 2008. EV71 subgenotype C2 was first detected in Australia in 1995 [38] and three years later, this predominant type circulating in Taiwan led to a devastating outbreak [39]. From the phylogenetic analysis of VP1 in the present study, two EV71 strains from Hong Kong, V08-2231530 and V08-2236079, detected in 2008 shared a high nucleotide sequence identity with other subgenotype C2 strains in recent years (2006–2008), forming a cluster separate from the subgenotype C2 strains discovered in the 1990s (Figure 4). This was in line with the result of a study showing that recent subgenotype C2 strains from the Netherlands and United Kingdom formed a clade distinct from a group of “old” strains detected in the Netherlands, Taiwan and the United States, with high bootstrap support of 100% [9]. Current classification of EV71 genotypes using VP1 gene sequences showed that the average nucleotide sequence divergences between and within subgenotypes were 10.1% and 3.6% respectively [19]. In the present study, the average nucleotide divergence between “old” and “new” subgenotype C2 strains was 4.6%, which lies between the two mean cut-off values, suggesting the emergence of a distinct lineage of subgenotype C2 strains in Hong Kong. The minority of EV71 subgenotype C2 strains detected only in 2008 in Hong Kong could be due to the importation of this subgenotype from neighboring countries, including mainland China [40]. Further epidemiological and genomic studies are required to identify the emergence of novel types of EV71 and assess their potential in causing epidemics in the near future.

To better understand the evolution of EV71, evolutionary rates and divergence dates of EV71 were also examined using VP1 gene sequence. VP1 gene was chosen because it shows a high degree of genetic diversity and no recombination has been mapped within VP1 gene [41], while recombination within 5'UTR, P2 and P3 regions is frequent in EV71 [15,17,22]. The evolutionary rate of EV71 was 3.1 × 10^-3^ s/s/y comparable to that of other enterovirus, such as EV68 (4.93 × 10^-3^ s/s/y) [42], but was lower than those of echovirus 30 (8.3 × 10^-3^ s/s/y) [43] and poliovirus (1.03 × 10^-2^ s/s/y for P1 region), with the latter shown to be the highest rate among picornaviruses [44]. Molecular clock analysis using VP1 gene sequences suggested that the tMRCA of all EV71 genotypes most likely emerged in the 1900s (mean: 1905), which was similar to that shown in a study from China (mean: 1911) [22], but was different from that demonstrated in another study (mean: 1941) [45], it is probably due to different sample sizes and model parameters used in the analyses. While the tMRCA of genotype B and C in the 1920s (mean: 1925), that of genotype B in the 1950s (mean: 1953), that of genotype A in the late 1960s (mean: 1967), and that of genotype C in the early 1970s (mean: 1972). A recent study has demonstrated the tMRCA for EV71 strains of subgenotypes C4a and C4b [22], but authors did not notice that recombination events have occurred in these strains, which can bias estimates of the tMRCA [46]. Although the tMRCA of the EV71 “double-recombinant” strains of “genotype D” could not be determined by molecular clock analysis, the detection of “genotype D” strains from Hong Kong and mainland China from year 1998 suggested that this genotype has emerged not later than this year. Since EV71 keeps on evolving via mutation and recombination [15,19], molecular surveillance is of vital importance in monitoring the genetic variations of circulating EV71 strains and the emergence of new types or recombinants of EV71.

## Conclusions

Though EV71 commonly causes mild diseases such as HFMD and herpangina in children, it can result in severe cardiorespiratory and neurological complications. EV71 “double-recombinant” strains of “genotype D” have been persistently circulating in Hong Kong over a decade, with “genotype D1b” strains being dominant in recent years. Furthermore, a distinct lineage of subgenotype C2 strains has emerged in Hong Kong in 2008. The generation of novel genotype resulting from mutation or recombination between different genotypes or subgenotypes of EV71 co-circulating in the same place over time may result in epidemics. Thus, development of rapid and sensitive molecular methods for the detection of EV71 strains in clinical samples is crucial for detecting genetic changes in EV71 and preventing outbreaks due to emergence of new virus variants.

## Methods

### EV71 strains

Among EV71 strains positive in clinical specimens during 2004–2010 in Hong Kong, two to five strains from the peak season of HFMD (April-June; one strain selected randomly from each month, except the two that could only be isolated in September 2005) for each year were selected, depending on the availability of EV71 isolates in each year. Thus, twenty two EV71 strains detected from throat and rectal swab, NPA and stool samples of 10 inpatients from 6 hospitals and 12 outpatients were included in the present study (Table 1). The viruses were successfully isolated using rhabdomyosarcoma (RD) cell line. Ethics approval (reference number: UW 04–278 T/600) was obtained from Institutional Review Board of the University of Hong Kong/ Hospital Authority Hong Kong West Cluster for this study.

### RNA extraction

Viral RNA was extracted from the EV71 isolates using EZ1 Virus Mini Kit v2.0 (QIAgen, Hilden, Germany). The RNA was eluted in 60 μl of AVE buffer and was used as the template for reverse transcription polymerase chain reaction (RT-PCR).

### RT-PCR and sequencing of partial VP2-VP3, 2C and 3D gene regions of EV71 and phylogenetic analysis

The RNA was converted to cDNA by a combined random-priming and oligo(dT) priming strategy using SuperScript III kit (Invitrogen, San Diego, CA, USA). The partial VP2-VP3, 2C and 3D gene regions of EV71 from the clinical specimens were amplified and sequenced using the primers shown in Additional file 3: Table S2 and the strategy described in our previous publication [15]. Phylogenetic trees of each region were constructed using maximum likelihood (ML) method in MEGA5 with the best models K2+G for partial VP2-VP3, K2+G+I for partial 2C and TN93+G for partial 3D [47]. The robustness of branches was assessed by bootstrap analysis with 1000 replicates.

### Complete genome sequencing of EV71

The complete genomes of eight EV71 strains (Table 1) were amplified and sequenced using the strategy described in our previous publications [10,15]. The RNA was converted to cDNA using a combined random-priming and oligo(dT) priming strategy. The cDNA was amplified by degenerate primers designed by multiple alignment of the genomes of EV71 of all genotypes and additional primers designed from the results of the first and subsequent rounds of sequencing. These primers sequences are shown in Additional file 4: Table S3. The 5′ ends of the viral genomes were confirmed by rapid amplification of cDNA ends using the 5′/3′ RACE kit (Roche, Germany). Sequences were assembled and manually edited to produce final sequences of the viral genomes.

### Genome analysis

The nucleotide sequences of the genomes and the deduced amino acid sequences of the single open reading frame (ORF) were compared to those of EV71 strains with available sequences in GenBank (Additional file 5: Table S4). The number of synonymous substitutions per synonymous site, Ks, and the number of non-synonymous substitutions per non-synonymous site, Ka, for each coding region was calculated using the Nei and Gojobori substitution model with Jukes-Cantor correction in MEGA5 [47]. Phylogenetic tree construction was performed using ML method in MEGA5 with the best models K2+G for 5′UTR and TN93+G+I for P1, P2 and P3 regions [47], with bootstrap values calculated from 1000 trees. Recombination analysis was conducted using a nucleotide alignment of the genome sequences of our EV71 strains, EV71 genotype A prototype strain BrCr (GenBank accession no. U22521), EV71 genotype B strain Nagoya (GenBank accession no. AB482183), EV71 genotype C strain 4643-TW98 (GenBank accession no. JN544418), and CVA16 prototype strain G-10 (GenBank accession no. U05876) was generated by ClustalX version 2.0 [48], and edited manually. Once aligned, similarity plot and bootscan analyses were performed using SimPlot version 3.5.1 (window size, 500 bp; step, 20 bp).

### Complete VP1 gene sequencing of EV71, phylogenetic analysis and estimation of divergence dates

The complete VP1 gene sequences of EV71 strains were amplified and sequenced using the primers shown in Additional file 3: Table S2. The PCR mixture (25 μl) contained DNA, iProof High-fidelity PCR buffer (Bio-Rad), 200 μM of each dNTP and 0.5 U iProof High-fidelity DNA polymerase (Bio-Rad). The mixtures were amplified by using 40 cycles of 98°C for 10 s, 55°C for 30 s and 72°C for 50 s, with a final extension at 72°C for 10 min, in an automated thermal cycler (Applied Biosystems). Phylogenetic tree of VP1 gene sequences (Additional file 5: Table S4) was constructed using ML method in MEGA5 with the best model K2+G+I [47], with bootstrap values calculated from 1000 trees. Divergence times for EV71 genotypes A, B and C were calculated using a Bayesian Markov Chain Monte Carlo (MCMC) approach as implemented in BEAST (Version 1.7.4) as described elsewhere [22,49]. Exponential growth population size coalescent was used as tree prior. Analysis was performed under the best substitution model GTR+I determined by ModelGenerator [50] for VP1 gene sequence data using a lognormal relaxed molecular clock. The MCMC analysis was run for 2 × 10^8^ generations and sampled every 1000 steps. Convergence was assessed on the basis of the effective sampling size after a 10% burn-in using Tracer software Version 1.5 [49]. The mean tMRCA and the highest posterior density regions at 95% (HPD) were calculated. The trees were summarized in a target tree by the Tree Annotator program included in the BEAST package by choosing the tree with the maximum sum of posterior probabilities (maximum clade credibility) after a 10% burn-in.

### Nucleotide sequence accession number

The complete genome sequences of the eight EV71 strains and the complete VP1 gene sequences of 22 EV71 strains (Table 1) have been deposited in the GenBank database under accession numbers KC436265 to KC436286.

## Abbreviations

EV: Enterovirus; HFMD: Hand foot and mouth disease; HEV-A: Human enterovirus species A; CVA16: Coxsackievirus A16; NPA: Nasopharyngeal aspirate; UTR: Untranslated region; s/s/y: substitutions per site per year; tMRCA: time to most recent common ancestor; RD: Rhabdomyosarcoma; RT-PCR: Reverse transcription polymerase chain reaction; ML: Maximum likelihood; RACE: Rapid amplification of cDNA ends; ORF: Open reading frame; MCMC: Markov chain monte carlo; BEAST: Bayesian evolutionary analysis by sampling trees; HPD: High posterior density.

## Competing interests

The authors declare that they have no competing interest.

## Authors’ contributions

CCYY, SKPL, PCYW and KYY designed the study. JYCL prepared virus isolates and clinical data. CCYY conducted experiments. CCYY and SKPL contributed to analysis and interpretation of data. CCYY, SKPL, JYCL, KHC, PCYW and KYY wrote the manuscript. All authors read and approved the final manuscript.

## Supplementary Material

Additional file 1: Table S1 Estimation of nonsynonymous and synonymous substitution rates in the seven genomes of EV71 subgenotype C4 (proposed genotype D). Click here for file

Additional file 2: Figure S1 Comparative sequence analysis of the 2A-2B junction (**A**). Multiple alignment of the nucleotide sequences of EV71 “genotype D” strain V10-2234054, EV71 genotype B strain Nagoya and genotype C strain 4643-TW98. In EV71 genotype B and EV71 genotype C, only the nucleotides different from those in EV71 strain V10-2234054 are depicted. The nucleotides in EV71 genotype C that are the same as those in EV71 strain V10-2234054 but different from those in EV71 genotype B are highlighted in grey, and those in EV71 genotype B that are the same as those in V10-2234054 but different from those in EV71 genotype C are highlighted in black. Comparative sequence analysis of the 3C region (**B**). Multiple alignment of the nucleotide sequences of EV71 strain V10-2234054, EV71 genotype B strain Nagoya and CVA16 strain G-10. In EV71 genotype B and CVA16 strain G-10, only the nucleotides different from those in EV71 strain V10-2234054 are depicted. The nucleotides in EV71 genotype B that are the same as those in EV71 strain V10-2234054 but different from those in CVA16 strain G-10 are highlighted in grey, and those in CVA16 strain G-10 that are the same as those in V10-2234054 but different from those in EV71 genotype B are highlighted in black. The predicted breakpoint position by bootscan analysis is indicated by an arrow. Click here for file

Additional file 3: Table S2 Primers used for PCR and sequencing of the partial VP2-VP3, 2C and 3D gene regions and the complete VP1 gene of the 22 EV71 strains. Click here for file

Additional file 4: Table S3 Primers used for complete genome sequencing on eight EV71 strains. Click here for file

Additional file 5: Table S4 List of EV71 strains used in the present study. Click here for file
