# Supplementary material for: Genetic characterization of EV71 isolates from 2004 to 2010 reveals predominance and persistent circulation of the newly proposed genotype D and recent emergence of a distinct lineage of subgenotype C2 in Hong Kong
Source: Virol J. 2013 Jul 4;10:222. doi: 10.1186/1743-422X-10-222 (PMC3716818; doi:10.1186/1743-422X-10-222)
Supplement: Additional file 1: Table S1 — Estimation of nonsynonymous and synonymous substitution rates in the seven genomes of EV71 subgenotype C4 (proposed genotype D). [file 1743-422X-10-222-S1.doc]

**Table S1. Estimation of nonsynonymous and synonymous substitution rates in the seven** genomes of EV71 subgenotype C4 (proposed genotype D)

| Gene | Ka | Ks | Ka/Ks |
| --- | --- | --- | --- |
| VP4 | 0.004 | 0.192 | 0.021 |
| VP2 | 0.002 | 0.213 | 0.009 |
| VP3 | 0 | 0.215 | 0 |
| VP1 | 0.003 | 0.206 | 0.015* |
| 2A | 0.004 | 0.256 | 0.016 |
| 2B | 0.003 | 0.172 | 0.017 |
| 2C | 0.003 | 0.258 | 0.012 |
| 3A | 0.008 | 0.249 | 0.032 |
| 3B | 0.023 | 0.114 | 0.202 |
| 3C | 0.005 | 0.201 | 0.025 |
| 3D | 0.008 | 0.248 | 0.032 |

*: Ka/Ks calculated from VP1 sequences of 20 EV71 strains of subgenotype C4 (proposed genotype D)
