# Supplementary figures and images for: Genetic characterization of EV71 isolates from 2004 to 2010 reveals predominance and persistent circulation of the newly proposed genotype D and recent emergence of a distinct lineage of subgenotype C2 in Hong Kong
Source: Virol J. 2013 Jul 4;10:222. doi: 10.1186/1743-422X-10-222 (PMC3716818; doi:10.1186/1743-422X-10-222)

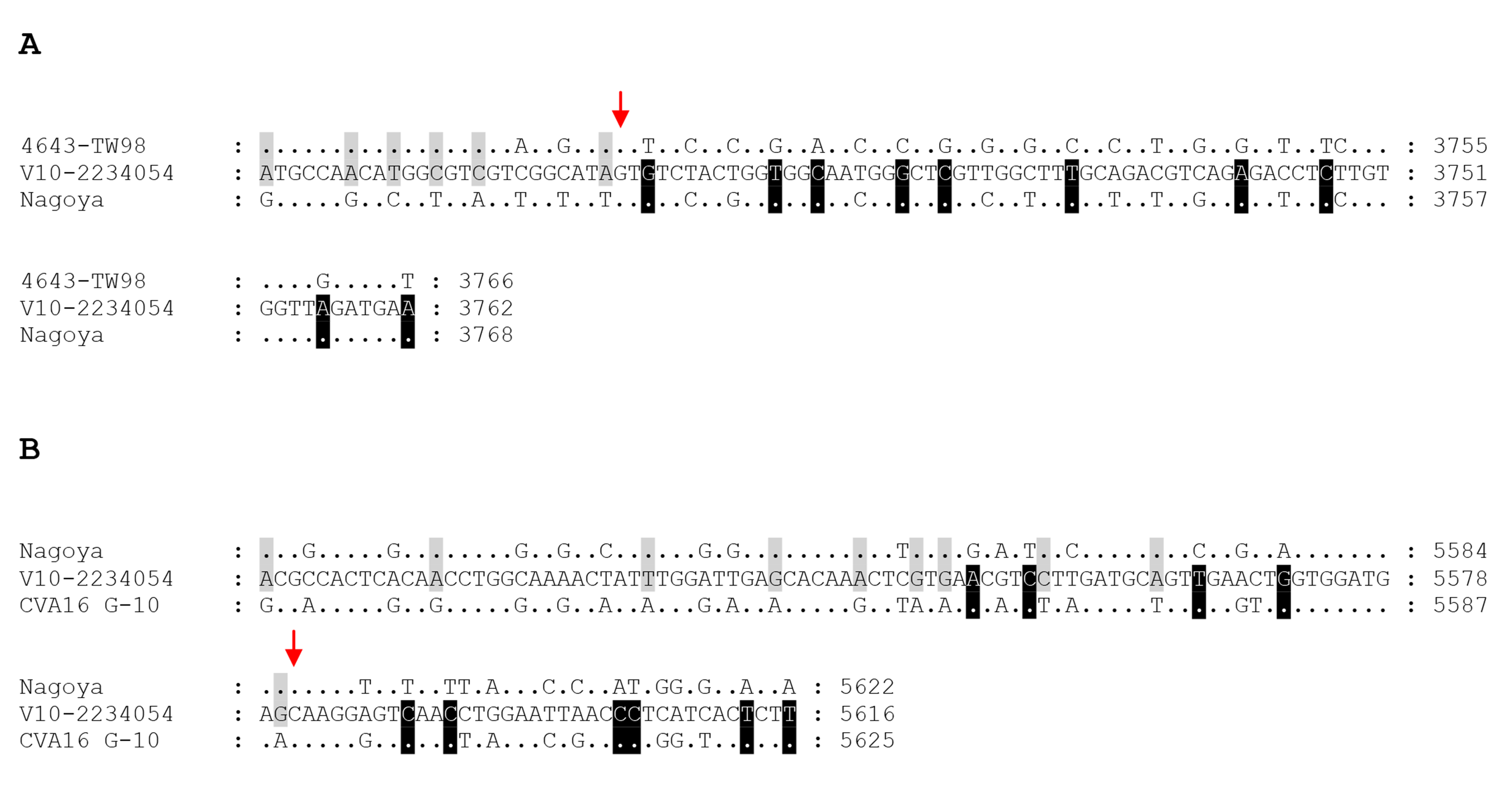

Supplement: Additional file 2: Figure S1 — Comparative sequence analysis of the 2A-2B junction (A). Multiple alignment of the nucleotide sequences of EV71 “genotype D” strain V10-2234054, EV71 genotype B strain Nagoya and genotype C strain 4643-TW98. In EV71 genotype B and EV71 genotype C, only the nucleotides different from those in EV71 strain V10-2234054 are depicted. The nucleotides in EV71 genotype C that are the same as those in EV71 strain V10-2234054 but different from those in EV71 genotype B are highlighted in grey, and those in EV71 genotype B that are the same as those in V10-2234054 but different from those in EV71 genotype C are highlighted in black. Comparative sequence analysis of the 3C region (B). Multiple alignment of the nucleotide sequences of EV71 strain V10-2234054, EV71 genotype B strain Nagoya and CVA16 strain G-10. In EV71 genotype B and CVA16 strain G-10, only the nucleotides different from those in EV71 strain V10-2234054 are depicted. The nucleotides in EV71 genotype B that are the same as those in EV71 strain V10-2234054 but different from those in CVA16 strain G-10 are highlighted in grey, and those in CVA16 strain G-10 that are the same as those in V10-2234054 but different from those in EV71 genotype B are highlighted in black. The predicted breakpoint position by bootscan analysis is indicated by an arrow. [file 1743-422X-10-222-S2.tiff]
