# Supplementary material for: Genetic characterization of EV71 isolates from 2004 to 2010 reveals predominance and persistent circulation of the newly proposed genotype D and recent emergence of a distinct lineage of subgenotype C2 in Hong Kong
Source: Virol J. 2013 Jul 4;10:222. doi: 10.1186/1743-422X-10-222 (PMC3716818; doi:10.1186/1743-422X-10-222)
Supplement: Additional file 3: Table S2 — Primers used for PCR and sequencing of the partial VP2-VP3, 2C and 3D gene regions and the complete VP1 gene of the 22 EV71 strains. [file 1743-422X-10-222-S3.doc]

**Table S2.** **Primers used for PCR and sequencing of the partial VP2-VP3, 2C and 3D gene regions and the complete VP1 gene of the 22 EV71 strains**

| Primer number | Primer sequence (5' - 3') | Genomic region (PCR product size) |
| --- | --- | --- |
| LPW7884 F | TGCCCACAYCARTGGATHAA | VP2-VP3 (569 bp) |
| LPW7885 R | CCTGACCACTGNGTRTARTA |
| LPW7890 F | AGGAGTGAYTAYGAYATHGT | 2B-2C (623 bp) |
| LPW7891 R | CCATCAAARTGRTCNGGRTC |
| LPW7900 F | GAACAAGGAGARATHCARTG | 3C-3D (455 bp) |
| LPW7901 R | AGACCATAYTTRTCCATRTA |
| LPW7875 F | CAGCCTATATAATAGCACTAGC | VP3-2A (1364 bp)* |
| LPW7877 R | AGTAGACACTATACCGACGA |
| LPW8005 F | CACTAGCTTAATGGAGAGACT | VP3-2A (1533 bp)^#^ |
| LPW21757 R | TCCCAAACAAGATTTGCCCA |

F, forward primer; R, reverse primer.

*: covering entire VP1 of subgenotype C4 (proposed genotype D)

^#^: covering entire VP1 of subgenotype C2
