# Supplementary material for: Genetic characterization of EV71 isolates from 2004 to 2010 reveals predominance and persistent circulation of the newly proposed genotype D and recent emergence of a distinct lineage of subgenotype C2 in Hong Kong
Source: Virol J. 2013 Jul 4;10:222. doi: 10.1186/1743-422X-10-222 (PMC3716818; doi:10.1186/1743-422X-10-222)
Supplement: Additional file 4: Table S3 — Primers used for complete genome sequencing on eight EV71 strains. [file 1743-422X-10-222-S4.doc]

**Table S3.** **Primers used for complete genome sequencing on eight EV71 strains**

| Virus strains | Primer number | Primer sequence (5' - 3') | Genomic region  (PCR product size) |
| --- | --- | --- | --- |
| EV71 subgenotype C4* |  |  |  |
| 04, 05, 06, 07, 08, 09, 10 | LPW21037 F | TTAAAACAGCCTGTGGGTTG | 5'UTR  (281-283 bp) |
|  | LPW8213 R | TCCGCAACTTTCATGGTGTT |
| 04, 05, 06, 07, 08, 09, 10 | LPW5218 F | CAAGCACTTCTGTBWCCCCGG | 5'UTR  (401 bp) |
|  | LPW5228 R | GAAACACGGACACCCAAAGTAGT |
| 04, 05, 06, 07, 08, 09, 10 | LPW14112 F | CCCCTGAATGCGGCTAATCC | 5'UTR-VP2  (1162 bp) |
|  | LPW21580 R | AGYGGGCTAATAGGYACAAC |
| 04, 05, 06, 07, 08, 09, 10 | LPW7884 F | TGCCCACAYCARTGGATHAA | VP2-VP3  (569 bp) |
|  | LPW7885 R | CCTGACCACTGNGTRTARTA |
| 04, 05, 06, 07, 08, 09, 10 | LPW21583 F | GTGTTYAGAGCYGAYCCYGG | VP3-2C  (2142 bp) |
|  | LPW8006 R | GTCATTGAACTTCTTGAGCCA |
| 04 | LPW7875 F | CAGCCTATATAATAGCACTAGC | VP3-2A  (1363 bp) |
|  | LPW7877 R | AGTAGACACTATACCGACGA |
| 05, 06, 08, 09 | LPW8053 F | CTGAGACCACTCTTGATAGT | VP1-2A  (1075 bp) |
|  | LPW8054 R | ACGTCTGCAAAGCCAACGA |
| 07 | LPW7875 F | CAGCCTATATAATAGCACTAGC | VP3-2A  (1048 bp) |
|  | LPW18459 R | TGTCGGTTTACCACTCTAAAG |
| 10 | LPW8053 F | CTGAGACCACTCTTGATAGT | VP1-2A  (727 bp) |
|  | LPW18459 R | TGTCGGTTTACCACTCTAAAG |
| 04, 05, 06, 07, 08, 09, 10 | LPW7890 F | AGGAGTGAYTAYGAYATHGT | 2B-2C  (623 bp) |
|  | LPW7891 R | CCATCAAARTGRTCNGGRTC |
| 04 | LPW14619 F | GCAGTTCAAGAGCAAACACCG | 2C-3D  (1646 bp) |
|  | LPW20256 R | CAGGTTCTTTGTTTCCCTCG |
| 05, 07, 08, 09, 10 | LPW14619 F | GCAGTTCAAGAGCAAACACCG | 2C-3D  (1612 bp) |
|  | LPW8012 R | TACACTAGGTTCTAACTTGGT |
| 06 | LPW14619F | GCAGTTCAAGAGCAAACACCG | 2C-3D  (1655 bp) |
|  | LPW7997 R | GCAAGACAGCTGGCTCCTTA |
| 04, 05, 06, 07, 08, 09, 10 | LPW7900 F | GAACAAGGAGARATHCARTG | 3C-3D  (455 bp) |
|  | LPW7901 R | AGACCATAYTTRTCCATRTA |
| 04, 05, 06, 07, 08, 09, 10 | LPW8040 F | TCTTCACACTAGTGCAGGTT | 3D-3'UTR  (1177 bp) |
|  | LPW418 R | GACCACGCGTATCGATGTCGACTTTTTTTTTTTTTTTTV |
| 04 | LPW14488 F | CAACTCAATGATTAACAACATC | 3D-3'UTR  (619 bp) |
|  | LPW418 R | GACCACGCGTATCGATGTCGACTTTTTTTTTTTTTTTTV |
| 05, 06, 07, 08, 09, 10 | LPW8052 F | CCCGATGAACAGTTTCCATT | 3D-3'UTR  (369 bp) |
|  | LPW418 R | GACCACGCGTATCGATGTCGACTTTTTTTTTTTTTTTTV |
|  |  |  |  |
| EV71 subgenotype C2  (strain V08-2236079) |  |  |  |
|  | LPW21037 F | TTAAAACAGCCTGTGGGTTG | 5'UTR  (464 bp) |
|  | LPW5225 R | TCCTCCGGCCCCTGAATG |
|  | LPW5218 F | CAAGCACTTCTGTBWCCCCGG | 5'UTR  (401 bp) |
|  | LPW5228 R | GAAACACGGACACCCAAAGTAGT |
|  | LPW14112 F | CCCCTGAATGCGGCTAATCC | 5'UTR-VP2  (1162 bp) |
|  | LPW21580 R | AGYGGGCTAATAGGYACAAC |
|  | LPW7884 F | TGCCCACAYCARTGGATHAA | VP2-VP3  (569 bp) |
|  | LPW7885 R | CCTGACCACTGNGTRTARTA |
|  | LPW21583 F | GTGTTYAGAGCYGAYCCYGG | VP3-2B  (2111 bp) |
|  | LPW21639 R | TGCTTCTGAGCCATCGGAAT |
|  | LPW21756 F | GCCGCTGAAATTGGAGCAT | VP1-2A  (848 bp) |
|  | LPW21757 R | TCCCAAACAAGATTTGCCCA |
|  | LPW7890 F | AGGAGTGAYTAYGAYATHGT | 2B-2C  (623 bp) |
|  | LPW7891 R | CCATCAAARTGRTCNGGRTC |
|  | LPW14619 F | GCAGTTCAAGAGCAAACACCG | 2C-3D  (1647 bp) |
|  | LPW20774 R | GCTGGCTCTTTGTTGCCCTT |
|  | LPW7900 F | GAACAAGGAGARATHCARTG | 3C-3D  (455 bp) |
|  | LPW7901 R | AGACCATAYTTRTCCATRTA |
|  | LPW21641 F | CTAGGCATCAAGAAGAGGGA | 3D-3'UTR  (1145 bp) |
|  | LPW418 R | GACCACGCGTATCGATGTCGACTTTTTTTTTTTTTTTTV |
|  | LPW21758 F | AAACAGGCAAGGAGTATGGT | 3D-3'UTR  (475 bp) |
|  | LPW418R | GACCACGCGTATCGATGTCGACTTTTTTTTTTTTTTTTV |

F, forward primer; R, reverse primer.

*For EV71 subgenotype C4 (proposed genotype D) strains, 04: V04-2218217; 05: V05-2243055; 06: V06-2218645; 07: V07-2231013; 08: V08-2221581; 09: V09-2225777; 10: V10-2234054.
