# Supplementary material for: Genetic characterization of EV71 isolates from 2004 to 2010 reveals predominance and persistent circulation of the newly proposed genotype D and recent emergence of a distinct lineage of subgenotype C2 in Hong Kong
Source: Virol J. 2013 Jul 4;10:222. doi: 10.1186/1743-422X-10-222 (PMC3716818; doi:10.1186/1743-422X-10-222)
Supplement: Additional file 5: Table S4 — List of EV71 strains used in the present study. [file 1743-422X-10-222-S5.doc]

**Table S4.** **List of EV71 strains used in the present study**

| Strain | Year of isolation | Place of isolation | Genotype* | Source | GenBank accession no. |
| --- | --- | --- | --- | --- | --- |
| BrCr | 1970 | USA | A | GenBank | U22521 |
| 001-Luan(CHN)-08 | 2008 | CHN | A | GenBank | GQ117124 |
| 5603-1965-NL | 1965 | NL | B0 | GenBank | AB491212 |
| 10076-1966-NL | 1966 | NL | B0 | GenBank | AB491213 |
| Nagoya | 1973 | JPN | B1 | GenBank | AB482183 |
| 2258-CA-79 | 1979 | USA | B1 | GenBank | AF135880 |
| 2952-SD-81 | 1981 | USA | B2 | GenBank | AF135888 |
| MS/7423/87 | 1987 | USA | B2 | GenBank | U22522 |
| SAR/SHA66 | 1997 | MAL | B3 | GenBank | AM396586 |
| 3799-SIN-98 | 1998 | SIN | B3 | GenBank | DQ341354 |
| 9/97/SHA89 | 1997 | MAL | B4 | GenBank | AJ586873 |
| 5666/sin/002209 | 2000 | SIN | B4 | GenBank | AF352027 |
| 5511-SIN-00 | 2000 | SIN | B5 | GenBank | DQ341364 |
| S19841-SAR-03 | 2003 | MAL | B5 | GenBank | DQ341363 |
| 6887-SYD-86 | 1986 | AUS | C1 | GenBank | AY722887 |
| 1M-AUS-12-00 | 2000 | AUS | C1 | GenBank | DQ341361 |
| 804/NO/03 | 2003 | NOR | C1 | GenBank | DQ452074 |
| 2641-AUS-95 | 1995 | AUS | C2 | GenBank | AF135947 |
| 2355-OK-97 | 1997 | USA | C2 | GenBank | AF135942 |
| 4643-TW98 | 1998 | TW | C2 | GenBank | JN544418 |
| Tainan/5746/98 | 1998 | TW | C2 | GenBank | AF304457 |
| 8M/AUS/6/99 | 1999 | AUS | C2 | GenBank | AF376109 |
| H0/6364/255/2006 | 2006 | UK | C2 | GenBank | AM939607 |
| 2527-2007-NL | 2007 | NL | C2 | GenBank | AB491219 |
| 265-2008-NL | 2008 | NL | C2 | GenBank | AB491220 |
| 97-56-CHN-97 | 1997 | CHN | C3 | GenBank | AB115494 |
| 03-KOR-2000 | 2000 | KOR | C3 | GenBank | DQ341356 |
| 06-KOR-2000 | 2000 | KOR | C3 | GenBank | DQ341355 |
| R13223-IND-01 | 2001 | IND | Unclassified | GenBank | AY179600 |
| 3254-TAI-98 | 1998 | TW | D1a | GenBank | AF286531 |
| SHZH98 | 1998 | CHN | D1a | GenBank | AF302996 |
| H25-CHN-00 | 2000 | CHN | D1a | GenBank | AB115492 |
| SHH02-17 | 2002 | CHN | D1a | GenBank | AY547500 |
| N3340-TW-02 | 2002 | TW | D1a | GenBank | EU131776 |
| THA-08-12242 | 2008 | THA | D1a | GenBank | FJ151494 |
| CQ03-1 | 2003 | CHN | D1b | GenBank | AY547501 |
| E2004104-TW | 2004 | TW | D1b | GenBank | DQ841964 |
| 540V/VNM/05 | 2005 | VNM | D1b | GenBank | AM490151 |
| SZ/HK08-5 | 2008 | CHN | D1b | GenBank | GQ279369 |
| 933V/VNM/05 | 2005 | VNM | C5 | GenBank | AM490161 |
| E2005125-TW | 2006 | TW | C5 | GenBank | EF063152 |
| V08-2231530 | 2008 | HK | C2 | This study | KC436282 |
| V08-2236079 | 2008 | HK | C2 | This study | KC436270 |
| V04-2216042 | 2004 | HK | D1b | This study | KC436273 |
| V04-2218217 | 2004 | HK | D1a | This study | KC436265 |
| V04-2223605 | 2004 | HK | D1b | This study | KC436274 |
| V05-2243055 | 2005 | HK | D1b | This study | KC436266 |
| V05-2243936 | 2005 | HK | D1b | This study | KC436275 |
| V06-2218645 | 2006 | HK | D1b | This study | KC436267 |
| V06-2223557 | 2006 | HK | D1a | This study | KC436276 |
| V06-2224881 | 2006 | HK | D1b | This study | KC436277 |
| V07-2231013 | 2007 | HK | D1b | This study | KC436268 |
| V07-2232477 | 2007 | HK | D1b | This study | KC436278 |
| V07-2233174 | 2007 | HK | D1b | This study | KC436279 |
| V08-2221581 | 2008 | HK | D1b | This study | KC436269 |
| V08-2228220 | 2008 | HK | D1b | This study | KC436280 |
| V08-2235341 | 2008 | HK | D1b | This study | KC436281 |
| V09-2220211 | 2009 | HK | D1b | This study | KC436283 |
| V09-2225777 | 2009 | HK | D1b | This study | KC436271 |
| V09-2229604 | 2009 | HK | D1b | This study | KC436284 |
| V10-2234054 | 2010 | HK | D1b | This study | KC436272 |
| V10-2240501 | 2010 | HK | D1b | This study | KC436285 |
| V10-2246239 | 2010 | HK | D1b | This study | KC436286 |

*: subgenotypes C4b and C4a proposed to be renamed as D1a and D1b respectively

Abbreviations: AUS, Australia; BRU, Brunei; CHN, China; JPN, Japan; KOR, Korea; HK, Hong Kong ; IND, India; MAL, Malaysia; NL, the Netherlands; NOR, Norway; SIN, Singapore; THA, Thailand; TW, Taiwan; UK, the United Kingdom; USA, the United States of America; VNM, Vietnam.
